# Localized Immune-Complex Small-Vessel Vasculitis Presenting as Unilateral Myositis After Hepatitis A

**DOI:** 10.7759/cureus.98584

**Published:** 2025-12-06

**Authors:** Mariam Zeidan, Rita Abou Zeid, Hajar Alhusani, Mira Merashli

**Affiliations:** 1 Faculty of Medicine, American University of Beirut, Beirut, LBN; 2 Rheumatology, American University of Beirut Medical Center, Beirut, LBN

**Keywords:** case report, hepatitis a, immune-complex vasculitis, mri-guided biopsy, myositis, small-vessel vasculitis, systemic inflammation, unilateral limb swelling, vasculitic myositis

## Abstract

Immune-complex small-vessel vasculitis (ICSVV) is a subtype of vasculitis characterized by inflammation and necrosis of small blood vessels caused by deposition of circulating immune complexes in vessel walls, followed by complement activation and recruitment of inflammatory cells. It typically targets postcapillary venules in the skin, but other small vessels in muscles, kidneys, gastrointestinal tract, and other organs can also be affected. Vasculitic myositis is usually bilateral and symmetric, with only a few reports of muscle-limited disease. Hepatitis A virus (HAV) is an uncommon trigger of vasculitis, in contrast to the well-recognized associations of hepatitis B with polyarteritis nodosa and hepatitis C with cryoglobulinemic vasculitis. We report a case of a woman in her early 30s who experienced intense discomfort in her left groin and thigh, accompanied by swelling and fever, two months after a confirmed acute HAV infection. Laboratory studies demonstrated severe systemic inflammation (WBC 30×10³/µL, C-reactive protein (CRP) 348 mg/L, erythrocyte sedimentation rate (ESR) >100 mm/h) with normal creatine kinase (CK). Axial fluorodeoxyglucose positron emission tomography/computed tomography (FDG-PET/CT) and coronal MRI revealed diffuse left-thigh inflammation without abscess. MRI-guided biopsy of the vastus lateralis demonstrated transmural inflammation and fibrinoid necrosis of intramuscular arteries with mixed CD4+/CD20+ infiltrates and C5b-9 deposition, consistent with ICSVV. The patient received oral prednisone 1 mg/kg daily (tapered over 10 weeks) and methotrexate 15 mg weekly; CRP decreased to 3.4 mg/L within four weeks. Remission was achieved by six months, with only mild residual numbness. This case represents the first report of biopsy-proven unilateral immune-complex vasculitic myositis temporally associated with HAV infection. It emphasizes the importance of considering vasculitic myositis in the differential diagnosis of focal limb swelling with systemic inflammation, even when CK levels are normal. It also highlights the role of MRI-guided biopsy in establishing the diagnosis. Early immunosuppressive therapy can improve outcomes in this rare but treatable condition.

## Introduction

Immune-complex small vessel vasculitis (ICSVV) is a subtype of vasculitis characterized by inflammation and necrosis of small blood vessels, including arterioles, capillaries, and venules, due to the deposition of immune complexes in the vessel walls [1]. While ICSVV most commonly targets postcapillary venules in the skin, the same immune complex deposition and complement activation can also occur in any organ, including the kidneys, gastrointestinal system, lungs, and intramuscular small vessels [2]. The clinical presentation ranges from cutaneous leukocytoclastic vasculitis to systemic disease with myositis, arthritis, or glomerulonephritis, depending on immune complex distribution [1].

Vasculitic myositis, a type of small-vessel vasculitis that only affects muscles, is uncommon and rare cases have been documented [2,3]. They typically appear with systemic inflammation (high-grade fever and elevated C-reactive protein (CRP) and erythrocyte sedimentation rate (ESR)) in addition to significant myalgia associated with weakness in the afflicted muscle groups [2,3]. After ruling out infection and paraneoplastic myopathy, the main diagnostic dilemma remains in differentiating ICSVV from idiopathic inflammatory myopathies (IIMs). Muscle involvement in previously reported cases of ICSVV is usually bilateral or symmetric, often affecting proximal muscle groups. In the largest series by Pinto et al., most patients exhibited proximal, symmetric weakness and systemic features, with only 12% having truly isolated muscle disease [4]. Khellaf et al. described 11 patients with calf-limited vasculitis, 80% of whom had bilateral muscle involvement [5]. Similarly, Benz et al. reported three cases of muscle-restricted small-vessel vasculitis, all of which had multifocal involvement including both proximal upper and/or lower extremities [2]. Unilateral muscle-limited vasculitis remains poorly described in the literature.

We present a woman in her early 30s with biopsy-proven ICSVV confined to her left thigh muscles, occurring two months after acute hepatitis A. She had severe unilateral thigh swelling and fever, without evidence of other organ involvement. To our knowledge, this is the first reported case of biopsy-proven unilateral immune-complex vasculitic myositis following hepatitis A virus (HAV) infection.

## Case presentation

A previously healthy woman in her early 30s presented to the rheumatology clinics with a one-week duration of intense left groin and thigh pain. The pain was constant and incapacitating, accompanied by a high-grade fever (up to 40°C), myalgias, and polyarthralgia. Notably, two months prior, she had documented acute hepatitis A infection. The patient denied any history of skin rash, photosensitivity, Raynaud’s phenomenon, oral or genital ulcers, sicca symptoms, joint swelling, morning stiffness, or family history of any autoimmune disease.

Upon examining her, the patient was febrile (39.5°C), tachycardic, and in visible distress. The left thigh was markedly swollen, erythematous, and tender, with inability to bear body weight. Tenderness was localized to the anterior compartment (quadriceps) and medial compartment (adductors), whereas the posterior (hamstring) compartment was non-tender. The distal pulses were palpable and equal. Neurologic examination showed patchy numbness over the medial thigh, without focal motor deficit or reflex changes.

Laboratory tests showed high inflammatory markers with leukocytosis and left shift. Routine chemistry, including liver enzymes, glucose, and creatine kinase (CK), was within normal ranges. Urinalysis was normal, with no hematuria or proteinuria. Creatinine was mildly elevated at 1.7 mg/dL but subsequently normalized on follow-up. These findings suggest a transient prerenal azotemia related to dehydration from reduced oral intake, as the patient was in severe pain. Hepatitis serologies showed positive HAV IgM and IgG (consistent with recent HAV) with negative HAV PCR; tests for hepatitis B surface antigen (HBsAg), hepatitis C antibody (HCV Ab), were negative. The remainder of the infectious workup, including blood cultures, Brucella, tuberculosis, and HIV serologies, was negative. Additionally, the autoimmune workup revealed no abnormalities. Autoantibodies, including antinuclear antibody (ANA); extractable nuclear antigen (ENA) panel comprising double-stranded DNA, Sjögren’s-syndrome-related antigen A (SSA), Sjögren’s-syndrome-related antigen B (SSB), Jo-1, and Smith antibodies; anti-neutrophil cytoplasmic antibody (ANCA); and rheumatoid factor (RF), were all negative. Chest X-ray showed no hilar lymphadenopathies, which decreases the likelihood of sarcoidosis. A transthoracic echocardiography was unremarkable. Serum IgG1 subclass was elevated (21 mg/dL, normal <10 mg/dL), indicating immune activation. Cryoglobulins were undetectable (no cryocrit). Serum complement levels revealed normal C3 but mildly reduced C4 (Table 1), consistent with modest classical-pathway consumption. Key laboratory results are summarized in Table 1.

**Table 1:** Key laboratory findings CRP, C-reactive protein; ESR, erythrocyte sedimentation rate; CK, creatine kinase.

Laboratory Test	Result	Normal Range
WBC count (×10^3^/µL)	30.0	4.0-11.0
CRP (mg/L)	348	<5
ESR (mm/h)	>100	<20
CK (U/L)	84	24-195
D-dimer (ng/mL)	4286	<500
Creatinine (mg/dL)	1.7	0.5-1.0
IgG1 subclass (mg/dL)	21	<10
C3 (mg/dL)	95	90-180
C4 (mg/dL)	8	10-40

**Figure 1:**
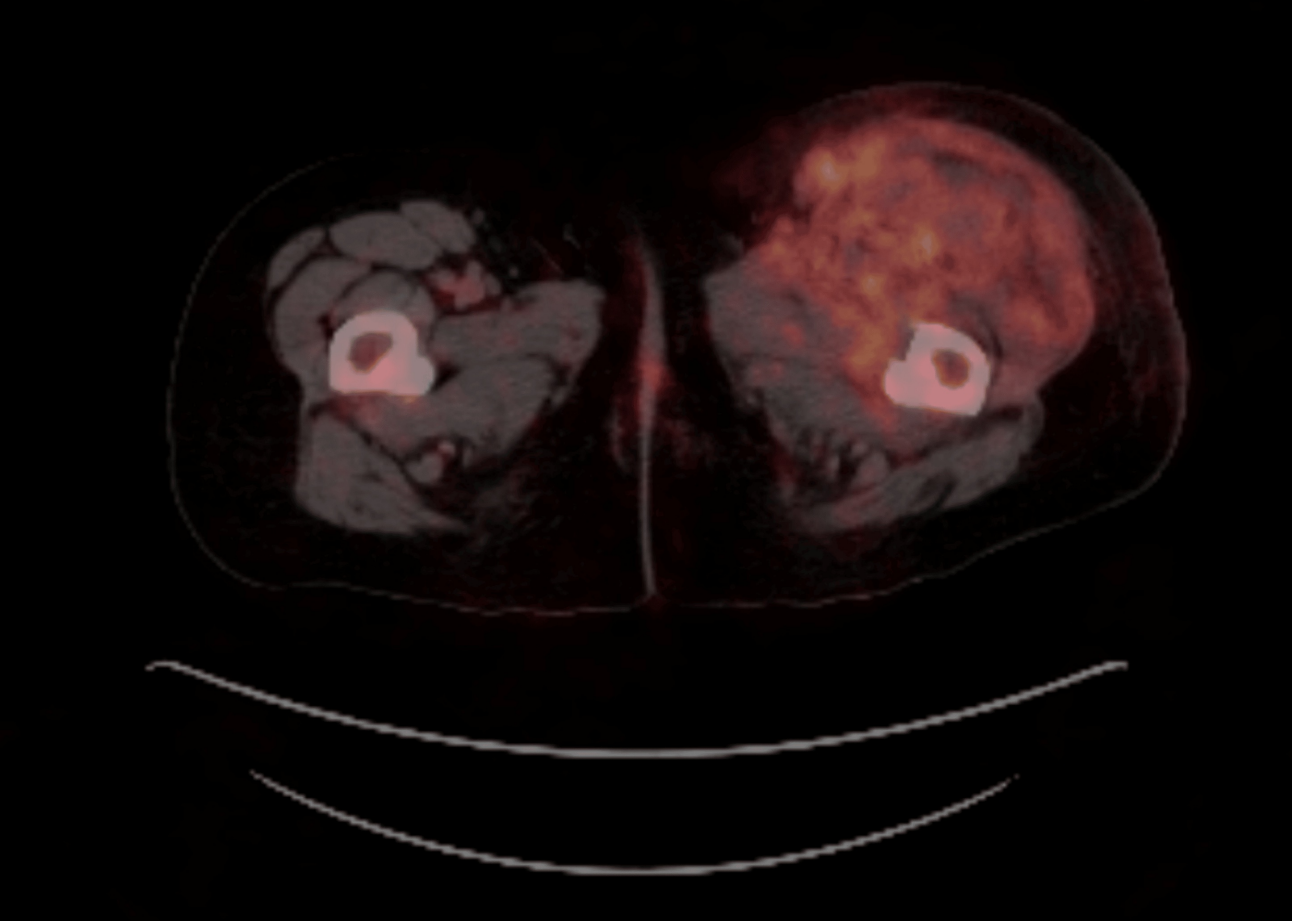
Axial FDG-PET/CT of the left thigh showing intense uptake in the quadriceps and adductor muscles, consistent with active inflammation. FDG, fluorodeoxyglucose; PET/CT, positron emission tomography/computed tomography.

Contrast computed tomography (CT) of the abdomen/pelvis showed left-sided psoas muscle and retroperitoneal fat stranding. A positron emission tomography-CT (PET-CT) revealed diffusely increased fluorodeoxyglucose (FDG) uptake throughout the left thigh muscle, including the quadriceps, adductors, and iliopsoas, with sparing of the hamstrings. It also demonstrated FDG uptake in the retroperitoneum, with no additional FDG-positive lesions except for a few subcentimetric reactive retrocrural lymph nodes (Figure 1).

**Figure 2:**
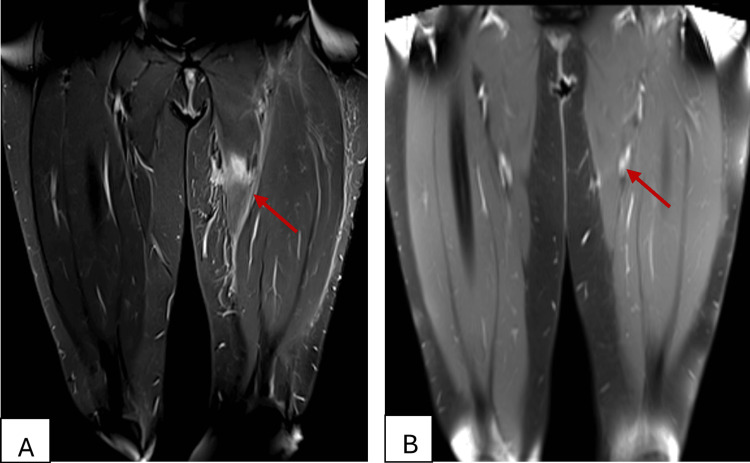
A: Coronal MRI of the left thigh (T2/STIR with contrast) demonstrating hyperintensity and enhancement in the quadriceps and adductor muscles without abscess or collection. B: Follow-up coronal MRI of the left thigh (T2/STIR with contrast) at 6 months showing complete resolution of muscle edema STIR, short-tau inversion recovery; MRI, magnetic resonance imaging.

A magnetic resonance imaging (MRI) was obtained to further delineate the retroperitoneal and muscular involvement. MRI showed evidence of T2 hyperintense muscle fibers of the quadriceps and adductor muscles with surrounding edema and enhanced post-IV injection of contrast material with no evidence of rupture, abscess, or collections seen (Figure 2A).

**Figure 3:**
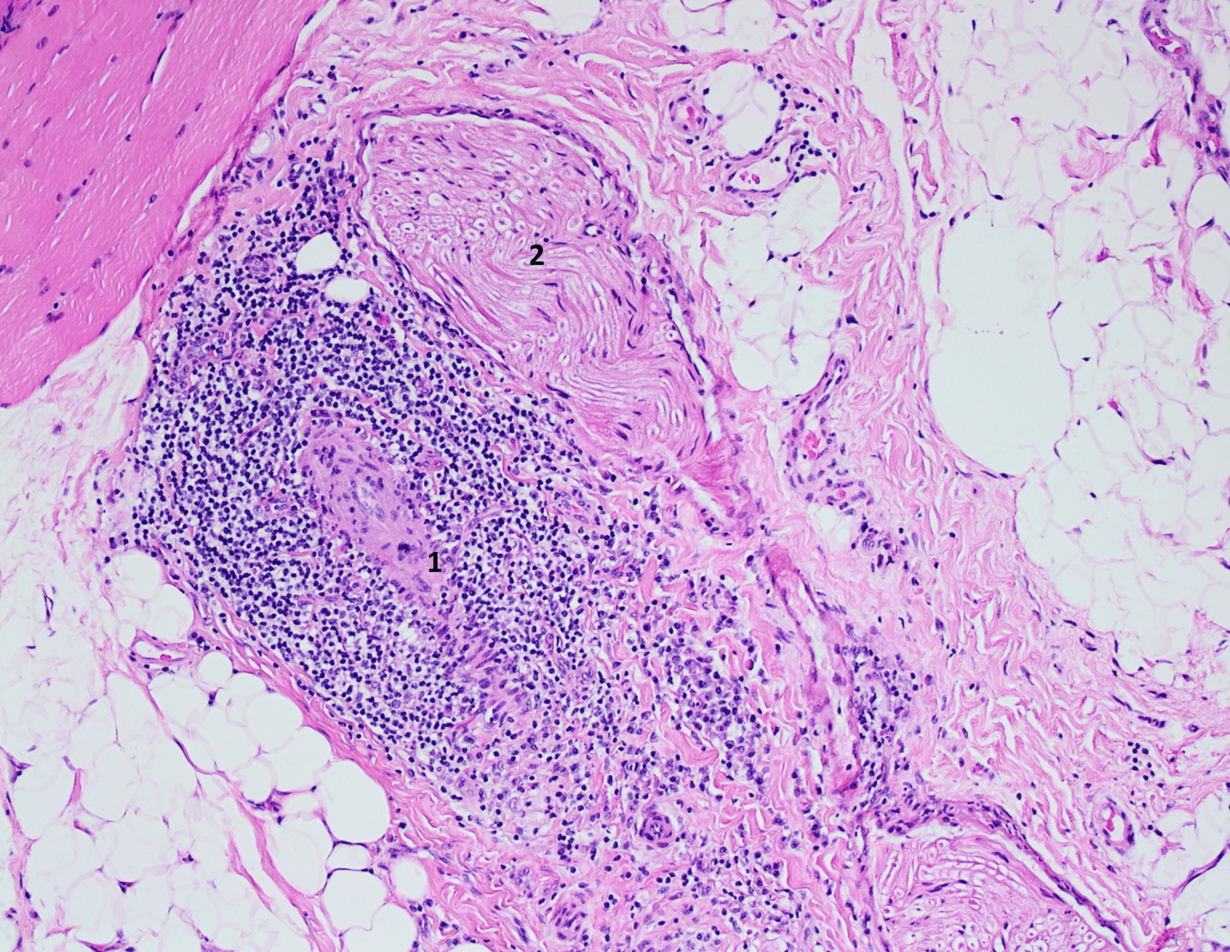
Hematoxylin and eosin (H&E) stain of skeletal muscle biopsy (×10, frozen section) showing 1. perivascular inflammatory infiltrates around small intramuscular arteries with thickened walls and mild myofiber size variation. 2. Normal-appearing nerve ending.

**Figure 4:**
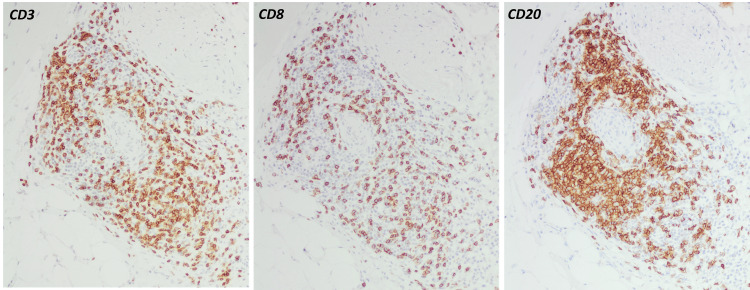
Immunohistochemical staining demonstrating perivascular infiltrates composed of mixed T (CD3⁺, CD8⁺) and B (CD20⁺) lymphocytes within the arterial wall. Inflammatory cells are negative for malignancy.

An MRI-guided biopsy of the left vastus lateralis was done. Histology revealed transmural inflammation and fibrinoid necrosis of small intramuscular arteries, as well as extensive lymphocyte cuffing (Figure 3). Immunohistochemistry indicated that the artery walls included mostly CD4+ T cells and CD20+ B cells, with MAC (C5b-9) deposition on the endothelium (Figure 4). There was no perifascicular atrophy or inclusion bodies, and skeletal muscle fibers exhibited little myopathic alteration (scattered tiny, angulated fibers) while maintaining overall architecture. Unlike inflammatory myositis, there was no substantial overexpression of major histocompatibility complex (MHC) class I on myofibers. No ragged-red fibers or mitochondrial inclusions were seen. Mild axonal loss and endoneurial fibrosis were seen in intramuscular nerve twigs, indicating a persistent neuropathic condition.

Given these results, the differential diagnoses were reviewed. We considered infectious myositis, given fever and raised inflammatory markers, but blood cultures were negative, and imaging showed no abscess or focal collection. Paraneoplastic syndromes were ruled out by the absence of occult malignancy on PET/CT scan. IMM was argued against because serum CK was normal in this acute phase, and myofiber necrosis was absent on histology. Sarcoidosis was also considered, as it can cause extrapulmonary or muscular inflammation; however, the chest X-ray showed no hilar lymphadenopathy, and the muscle biopsy demonstrated no noncaseating granulomas or sarcoid-type perivascular inflammation, findings that essentially exclude sarcoid myopathy. Systemic vasculitis was considered improbable with no organ involvement and negative ANCA serology. Cryoglobulinemic vasculitis was also unlikely since cryoglobulins were undetectable and C4 was only mildly low. In sum, clinical, radiologic, and pathologic evidence led to the diagnosis of localized ICSVV myositis, limited to the left thigh, temporally associated with recent HAV infection.

The patient was first treated empirically with broad-spectrum antibiotics while awaiting completion of the infectious and rheumatologic workup, but this proved ineffective. Then, she was started on a high dose of corticosteroids (prednisone 1 mg/kg per os (PO) daily), resulting in partial fever alleviation and a decrease in edema within days, although severe myalgias continued. Prednisone was tapered gradually by 5 mg per week over 10 weeks. Methotrexate 15 mg weekly PO, along with folic acid 5 mg weekly, was initiated concurrently as steroid-sparing immunosuppression. Within four weeks, the patient’s CRP dropped from 348 mg/L to 3.4 mg/L, and her pain and mobility significantly improved.

At the six-month follow-up, the patient had made a significant recovery. She reported only mild residual numbness in her medial thigh. Muscle strength was optimal, and gait was normal. Repeat electromyography and nerve conduction studies revealed no signs of myopathy or neuropathy. An MRI of the left thigh revealed that the edema had been resolved (Figure 2B). Laboratory results were near-normal: ESR 33 mm/h, CRP 3.4 mg/L, and CK 84 U/L. The patient continues to take low-dose methotrexate maintenance with no recurrence for one year.

## Discussion

Vasculitic myositis is an uncommon type of muscle inflammation characterized by necrotizing vasculitis of intramuscular arteries and subsequent ischemic myopathic alterations [6]. Unlike classic autoimmune myositis, the inflammation here is secondary to vascular injury rather than a primary immune attack on muscle fibers, which explains why CK levels are often normal or mildly elevated [6]. Published series commonly report that patients may experience proximal, symmetric weakness and myalgia while having normal or slightly raised CK values [4,5]. However, our patient had an unusual unilateral thigh-limited presentation following a recent HAV infection.

Chronic hepatitis viruses are known triggers for systemic vasculitis. Hepatitis B virus (HBV) is classically associated with polyarteritis nodosa (PAN), and hepatitis C virus (HCV) is linked to mixed cryoglobulinemia and ICSVV [7,8]. Hepatitis A has been rarely associated with small-vessel vasculitis or cryoglobulinemia [9]. A case has been reported of an eight-year-old child who developed cryoglobulinemic vasculitis following HAV infection and demonstrated a favorable response to oral corticosteroid therapy [10]. Our case broadens this spectrum to include isolated vasculitic myositis.

The presumed mechanism is thought to involve chronic immune system stimulation by the virus, resulting in the production of autoantibodies and the formation of circulating immune complexes. Deposition of these complexes in intramuscular vessel walls triggers complement activation and MAC (C5b-9) deposition, endothelial injury, fibrinoid necrosis, and downstream ischemic damage to adjacent myofibers. Vasculitic manifestations of viral hepatitis can appear within a few weeks during the acute phase (e.g., IgA-mediated vasculitis after HAV), several weeks or months in HBV-associated syndromes like PAN, and even during chronic or inactive infection phases [11]. Nomura et al. found C5b-9 (MAC) deposition on vessels in over 70% of vasculitic myopathy specimens, indicating immune-complex vasculitis [12]. This mechanism is consistent with our biopsy findings of MAC deposition and mixed CD4+/CD20+ perivascular infiltrates.

Laboratory results in vasculitic myositis frequently differ from those in primary inflammatory myositis. Published series often show normal or modestly increased CK values [2,4]. Pinto et al. noted increased CK in only 13% of patients, indicating an ischemia rather than myopathic disease of muscle fibers [4]. Similarly, Benz et al. noted that in their three cases, CK was not consistently elevated [2]. Our patient's CK was normal despite severe weakness, which corroborated these findings. Serum aldolase levels are often high in cases of tissue ischemia [4], but this was not checked in our patient. Benz et al. reported "highly elevated acute phase reactants" [2], and our patient also had high CRP. In general, myositis-specific autoantibodies are uncommon in localized or vasculitic myositis, consistent with our patient’s negative serology. ANCA-associated myositis is extremely rare and typically accompanies systemic vasculitis. In our patient, the absence of systemic organ involvement, together with negative ANCA and other autoimmune markers, supports a diagnosis of isolated immune-complex small-vessel vasculitis confined to the muscle.

Imaging plays a crucial role in diagnosis and monitoring, and can guide targeted biopsy. T2/short-tau inversion recovery (STIR) hyperintensity and contrast enhancement, which indicate edema and hypervascularity, are typically seen on MRI of the afflicted muscle. According to Khellaf et al., MRI was "the cornerstone of the diagnosis", revealing a hyperintense signal in the calf muscles that were involved [5]. Shimojima et al. also reported that MRI (T2/STIR) can help target biopsies by sensitively identifying areas of active vasculitis through hypervascular changes [13]. Consistent with previous accounts, our patient's MRI revealed active inflammation in the affected limb, delineated the extent of disease, and guided biopsy.

Muscle biopsy is a crucial diagnostic tool in evaluating vasculitic myositis. It confirms the diagnosis, helps distinguish between different types of myositis, assesses disease activity, guides therapeutic decisions, and provides valuable prognostic information. Pinto et al. found perivascular inflammation in all biopsies and transmural vessel-wall destruction in 86% of cases, including fibrinoid necrosis (62% of cases) [4]. Many specimens showed T cell-predominant (CD4+) infiltrates and complement deposition on vessel walls [4]. Immune-mediated necrotizing myopathy, by contrast, only rarely exhibits vascular complement deposits. On the other hand, medium-vessel vasculitis (such as PAN) or ANCA-associated vasculitis can be pauci-immune. Indeed, Benz et al. reported three patients with primary muscle-limited small-vessel vasculitis (microscopic-polyangiitis-type) who all had high inflammatory markers and myalgias [2]. A muscle biopsy of those patients showed fibrinoid vasculitis and focal infarct-like necroses, with complement (MAC) staining emphasizing vessel damage [2]. Similarly, Khellaf et al. examined 11 individuals with calf-limited vasculitis and discovered that 40% had medium-vessel (PAN-type) necrotizing vasculitis and 60% had leukocytoclastic (immune-complex) vasculitis upon biopsy [5]. In our patient, the pathology was confined to small intramuscular arteries with transmural necrosis, a mixed CD4+ T-cell and CD20+ B-cell perivascular infiltrate, and widespread C5b-9 deposition, findings that place this case at the immune-complex (“microscopic”) end of the spectrum described by Benz and Khellaf and distinguish it from pauci-immune medium-vessel or ANCA-associated processes [2,4,5].

There is no universally accepted treatment regimen for vasculitic myositis, primarily because of its rarity and clinical complexity. Management is individualized and guided by disease severity, systemic involvement, and underlying etiology, rather than following a standardized protocol. Typically, treatment involves a combination of glucocorticoids, immunosuppressive agents, with biologic therapies reserved for selected cases, all tailored to the patient’s specific disease subtype and severity. Pinto's study found that treatment with high-dose corticosteroids and steroid-sparing medicines improved 24 out of 25 patients, with an 84% five-year survival rate [4]. Khellaf et al. also found steroids beneficial for induction, although more than half (54%) needed further immunosuppression owing to relapses [5]. Benz et al. found that one of three patients attained remission with steroids alone, but the others required further treatment [2]. Isolated muscle-limited ICSVV appears to have a more favorable course compared to systemic vasculitis, with better survival and higher remission rates when treated early with corticosteroids and immunosuppressive agents [4-6]. Nevertheless, relapses are common in published cohorts, and long-term monitoring is crucial [4,5]. In our patient, prompt treatment with prednisone and methotrexate led to rapid clinical and laboratory remission, consistent with an immune-complex vasculitis that is responsive to therapy. The favorable outcome here echoes prior reports that muscle-limited vasculitis can be a curable condition if treated early. Given the high relapse rates reported in case series and the absence of randomized trials or formal guidelines for muscle-limited ICSVV, continuation of methotrexate as maintenance therapy, with close clinical and laboratory surveillance, was considered appropriate.

## Conclusions

To our knowledge, this report describes the first case of biopsy-proven unilateral immune-complex vasculitic myositis temporally associated with acute HAV infection, broadening the clinical scope of muscle-limited small-vessel vasculitis. Presentation included focal limb swelling, severe pain, markedly elevated inflammatory markers, normal CK values, and MRI findings of muscle edema. Early MRI-guided biopsy and immunosuppression resulted in a favorable outcome for this patient. Although HAV may have been the trigger, causality remains unproven. Clinicians should consider viral triggers beyond HBV/HCV when evaluating localized vasculitic myositis. Further research is needed to aid in rapid diagnosis and clarify long-term prognosis, relapse risk, and optimal therapy in this rare but curable condition.
